# Train-Induced Vibration Monitoring of Track Slab under Long-Term Temperature Load Using Fiber-Optic Accelerometers

**DOI:** 10.3390/s21030787

**Published:** 2021-01-25

**Authors:** Jianxiang Zhang, Wenzhu Huang, Wentao Zhang, Fang Li, Yanliang Du

**Affiliations:** 1 State Key Laboratory of Transducer Technology, Institute of Semiconductors, Chinese Academy of Sciences, Beijing 100083, China; zjxiang@semi.ac.cn (J.Z.); hwzhu@semi.ac.cn (W.H.); lifang@semi.ac.cn (F.L.); 2 College of Materials Science and Opto-Electronic Technology, University of Chinese Academy of Sciences, Beijing 100049, China; 3 College of Civil and Transportation Engineering, Shenzhen University, Shenzhen 518060, China; du_yanliang@szu.edu.cn

**Keywords:** optical fiber accelerometer, high-speed railway, ballastless track, long-term monitoring, wavelet packet energy, deformation

## Abstract

The train-induced vibration response provides a flexible solution for the real-time monitoring deformation of high-speed railway track slab in actual operation. This paper proposes a long-term real-time monitoring method for track slab deformation based on wavelet packet energy (WPE) using fiber optic accelerometers to record train-induced vibration. We found that the vibration response law of track slab deformation could be established by using the WPE of the frequency band covering the first- and second-order frequencies induced by the adjacent carriages. A field test was carried out for more than one year on the Beijing–Shanghai high-speed railway to investigate the train-induced vibration response law of track slab that was continuously deformed under a long-term temperature load. The maximum values of the WPE characteristic index appeared in winter and summer, and they were positively correlated with the temperature difference between the air environment and the track slab under the daily temperature load. These results were demonstrated to be consistent with the track slab deformation law for long-term and daily temperature loads. The novel method based on fiber optic accelerometers and WPE provides a new method for the long-term and real-time monitoring of track slab deformation.

## 1. Introduction

Ballastless track is a track structure that guarantees the reliable operation of high-speed railways due to its high smoothness, high stability, and low maintenance [1]. When subjected to temperature and trainloads [2,3], ballastless track slab structures generate deformation. As the operating time increases, the deformation of track slab will evolve into various types of damage, such as warping [4], cracking [5], and disengagement [6,7,8]. Once these types of damage reach a certain degree, they will seriously affect the structural stability and service performance of high-speed railways [9]. Therefore, the long-term and real-time monitoring of ballastless track slab deformation is of great significance for ensuring safe train operation.

At present, several studies on the deformation of ballastless track slabs have been carried out with various methods, such as surface and end angle displacement [10,11] and surface strain [12,13]. However, it is difficult to use these methods to detect internal and global deformations. The train-induced vibration response monitoring method [14,15] is based on the principle that deformation alters both the physical properties as well as the dynamic characteristics of track slab [16]. This method is not limited by the working time, and it does not affect the train operation, so it can be considered a real-time and global non-destructive monitoring technique [17]. Recently, several studies on the deformation of ballastless track slabs have been carried out based on train-induced vibration. Song [10] analyzed the relationship between the acceleration amplitude and deformation of track slab based on finite-element models and found that vertical acceleration response indexes of track slab were intensified at a certain level when deformation occurred. Wang [18] analyzed the influence of track slab disengagement on the vibration frequency spectrum. The track slab vibration frequency spectrum produced an obvious peak in the low-frequency part, and the amplitude was much larger than the normal acceleration spectrum curve. Zhu and Cai [19] investigated the interface damage between track slab and a cement asphalt (CA) mortar layer with temperature change and its influence on the accelerations of the track slab. The maximum vertical acceleration amplitude was about 3.2 times as large as that without damage.

The above simulation research on the vibration characteristics of deformed track slab has provided the research foundation and references needed for the current study. If a long-term monitoring test on the actual operation of high-speed railways could be carried out, it would perfect the deformation research of track slab. Unfortunately, during high-speed railway operation, the electromagnetic interference (EMI) of traditional vibration sensors is aggravated by electrification, power electronics, and magnetic breaks [20], which limits the long-term and real-time monitoring of train-induced vibration responses.

This paper proposes a long-term and real-time monitoring method for deformed track slabs based on wavelet packet energy (WPE) using fiber optic accelerometers to record train-induced vibration. The WPE of the frequency band covering the first- and second-order frequencies induced by adjacent carriages were used to establish the vibration response law of track slab deformation. Based on this method, we investigated the train-induced vibration response law of track slab that was continuously deformed under long-term temperature load. A field test was carried out on the Beijing–Shanghai high-speed railway in China for more than one year. The WPE characteristic index variations were consistent with the trend of track slab deformation under long-term and daily temperature loads. The new method based on WPE and fiber optic accelerometers is promising for the long-term and real-time monitoring of track plate deformation.

## 2. Methods

### 2.1. Train-Induced Vibration Signal Acquisition System

**Figure 1:**
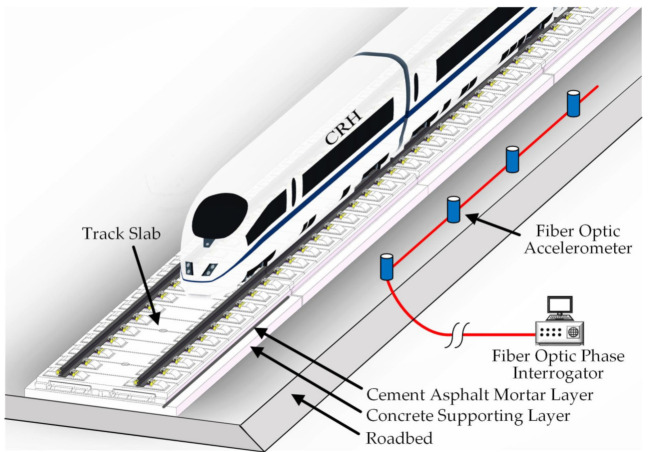
Composition of the experimental system.

The configuration of the long-term, real-time train-induced vibration signal acquisition system for ballastless track slab is shown in Figure 1. This system mainly consisted of four parts: high-speed trains, China Railway Track System (CRTS) II track slab, a fiber optic accelerometer array, and a phase interrogator.

High-speed trains acted as the excitation source of the system. The CRTS II track slab was laid on a high-speed railway with a high embankment, which was a laminated structure that mainly consisted of a rail and fastener system, concrete track slab, cement asphalt (CA) mortar layer, concrete supporting layer, and roadbed from top to bottom [6]. These track structure components formed a complete mechanic transfer system that evenly transferred the train-induced vibration to the base structure from top to bottom. Under the condition of track slab deformation, the integrity of the force transmission system of this track structure is expected to deteriorate over time, thereby causing the distribution of the vibration in the track structure to change.

The fiber optic accelerometer array is based on the unbalanced Michelson interferometer. The principle and performance of the sensor were reported in our previous study [21]. Inside the fiber optic accelerometer structure, two clamped metal diaphragms with the supported mass and movable lid constitute the sensing element of the accelerometer. The optical fiber sensing arm of the Michelson interferometer is wrapped around the surface of the upper moving lid and the lower fixed lid with a certain prestress. When the sensor is accelerated, the supported mass will have a displacement relative to the base and make the metal diaphragm deform, thus causing compression or extension of optical fibers wrapped around the surface of the upper and lower lids. This effect produces a change in the length of optical fibers, leading to a phase shift in the optical fiber interferometer. The fiber optic accelerometer array was arranged on the ballastless track roadbed to monitor train-induced vibration, taking into account its size and the requirements for the safe operation of the train [22]. According to the vertical dynamic coupled model of vehicle and double-block ballastless tracks with roadbed cracks [23] and the experimental investigation in [19], the train-induced vibration response recorded by this method of arranging sensors could be used to analyze the structural characteristics of track slab.

The phase interrogator was based on phase generated carrier (PGC), which is mainly composed of fiber laser, couple, photodetectors (PDs), analog-to-digital convertor, field-programmable gate array (FPGA) [24]. A narrow-linewidth fiber laser modulated by a digital to analog converter driven by FPGA with a sine wave. A space-division multiplexing technique using a 1 × 8 couple is used to extend the channel number of the system to achieve a long-distance, distributed measurement. The interferometric signals are received by PDs. The electrical output of the photodetectors is digitalized using a multichannel analog-to-digital convertor. The phase demodulation algorithm is accomplished on an FPGA board, and finally, acceleration is extracted. The fiber optic phase interrogator was connected with a fiber-optic accelerometer array through optical fibers to achieve phase to vibration acceleration conversion and data storage in real time.

The entire optical fiber sensing system could record vibration signals at a frequency of up to 500 Hz, which was sufficiently high to record the vibration response induced by high-speed trains made in China at a speed of about 300 km/h [25]. The data recording time was more than one year, covering the entire annual temperature change cycle to achieve the vibration response monitoring of the continuous deformation track slab under a long-term temperature load.

### 2.2. Vibration Response Analysis Based on WPE

With the alternation of daily and seasonal temperature loads, temperature stress undergoes tension–compression cycles, which causes the track slab to delaminate or even break [5]. Such damage has great effects on the dynamic responses of the track slab [19]. The deformation of the track slab weakens its bond with the CA mortar layer, leading to the attenuation of the vibration generated by the rail, which is caused by the train excitation being reduced when it is transmitted down to the track slab. This, in turn, causes the acceleration amplitude in the low-frequency range of the track slab to increase. Moreover, there is a certain gap between track slab and the CA mortar layer, about 0.1 mm to 2 mm [26]. When the train wheels pass by, the suspended part of the track slab is excited to produce low-frequency vibration. Therefore, the vibration of track slab in the low-frequency range is greatly increased. This leads to an increase in the signal energy in the low-frequency band [18].

In this research, we used WPE to analyze the train-induced vibration signal of a ballastless track slab under a temperature load. The WPE used a rich library of redundant bases [27] with arbitrary time-frequency resolution, including Biorthogonal, Coiflet, Harr, Symmlet, Daubechies, etc. [28]. Additionally, the complex structure of a raw signal was converted into a simple energy structure in the frequency domain [27,29], notably reducing the quantity of information but conserving the information related to the structure under testing. A wavelet packet is a generalization of a wavelet in that each octave frequency band of the wavelet spectrum is further subdivided into finer frequency bands by repeatedly using the two scale relations [30]. After the wavelet packet decomposition of the train-induced vibrations signal *s*(*t*), the total energy *E_T_* of *s*(*t*) could be obtained as follows:

(1) $$E=\sum_{i=1}^{2^{j}}\int_{-\infty }^{\infty }s_{j}^{i}{\left(t\right)}^{2}dt=\sum_{i=1}^{2^{j}}E_{i},$$

where *s* $s_{j}^{i}\left(t\right)$ is the reconstruction signal after the wavelet packet decomposition, *j* is the scale index, and *i* is the frequency index. It can be seen from Formula (1) that the total energy of the signal is the sum of the energies of the components of the wavelet packet in different frequency bands. The energy ratio of each frequency band to the total energy *R_i_* is generally used as the representation of the WPE energy:

(2) $$R_{i}=\frac{E_{i}}{E}.$$

**Figure 2:**
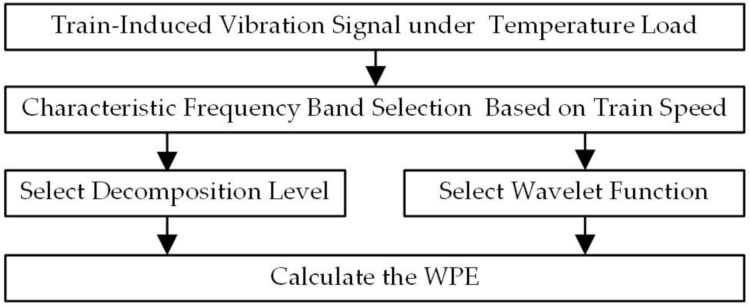
The wavelet packet energy (WPE) analysis process of the vibration response.

The vibration response of the high-speed railway track plate for the temperature load WPE analysis process is shown in Figure 2.

For the train-induced vibration response of the high-speed railway track slab, the periodic excitation frequencies under the repeated action of the vehicle axle loads have a relationship with the fundamental frequencies *f*_1_, *f*_2_, *f*_3_, and *f*_4,_ which are defined as shown in Formula (3) [25,31]:

(3) $$f_{i}=\frac{v}{L_{i}},i=1,2,3,4,$$

where *f*_i_ is the characteristic frequency of the ballastless track vibration generated by the train (Hz), *v* represents the train speed (m/s), and *L_i_* is the characteristic length of the train.

For the type of high-speed train passing through the test section in this study, the wheelbase *L*_1_ was about 2.5 m, the center distance of the adjacent bogies between the forward car and the backward *L*_2_ was about 7.5 m, the length between two bogie centers in one car *L*_3_ was about 17.5 m, and the center distance of two neighboring carriages *L*_4_ was about 25 m. The dominant frequencies were from the fundamental carriage length frequency *f*_4_, while *f*_1_, *f*_2_, and *f*_3_ provided a modulation amplitude effect [32,33]. Therefore, it was critical to select the characteristic frequency band of the WPE according to the train speed, which was necessary to avoid the modulation effect of *f*_1_, *f*_2_, and *f*_3_.

We selected the decomposition level according to the vibration signal sampling rate and the characteristic frequency band to obtain the highest possible frequency resolution of each package. To calculate the wavelet packet transform (WPT) energy, choosing the wavelet function was critical. In this work, we selected the Daubechies 6 wavelet function, which has been applied with very good results in railway applications, such as those in references [34,35]. Finally, we used the selected decomposition level and wavelet function to perform the wavelet packet decomposition of the vibration signal and to calculate the WPE.

## 3. Field Test

From 23 July 2019 to 29 September 2020, measurement of the train-induced vibration of track slab on the Beijing–Shanghai high-speed railway was performed. The measurement site was located near the Qufu East station. During the field experiment, the air temperature range of the test station was −10 °C to 35 °C, and the track slab temperature range was −15 °C to 50 °C. Many types of high-speed trains made in China, including the CRH380A (8 carriage formation), CRH380AL (16 carriage formation), and CRH380BL (16 carriage formation) trains [25], were adopted in this experiment, and the running speeds in the test section ranged from 120 km/h to 320 km/h.

At the test station, each slab unit had dimensions of 6.50 m × 2.55 m × 0.2 m (length × width × height), and pairs of rail platforms were arranged above with a spacing of 0.65 m. There were 50 mm wide joints between the track slabs, which were connected by six steel bars that extended from the ends of the slabs during the longitudinal connection, and the joints were poured with concrete. The adjustment layer of the CA mortar poured between the track slab, and the support layer had a thickness of 30 mm and the same width as the upper track slab. Its main function was to ensure the reliable bonding of the track slab and the support layer. The size of the concrete supporting layer was 2950 mm × 300 mm, and it was a continuous plate-like structure that was cast on-site.

**Figure 3:**
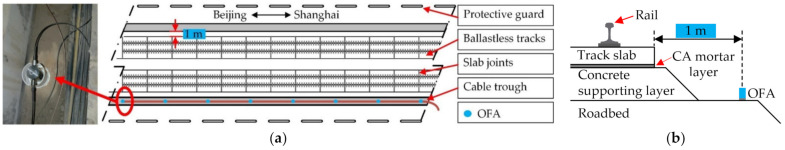
Arrangement of optical fiber accelerometers. (**a**) Top view. (**b**) Cross-section view. OFA (Optical fiber accelerometer); CA (cement asphalt).

The fiber optic accelerometers were in the K532 + 809 position of the Beijing–Shanghai high-speed railway, and they were glued to the bottom surface of the clean cable trough with marble adhesive (Hercules, manufactured by Wuhan Keda Marble Protective Materials Co., Ltd., Wuhan, China. E-modulus: ≥3000 MPa), which thickness is about 0.5 mm to 1 mm, as shown in Figure 3. The distance between the cable trough and the ballastless track was 1 m. To avoid the impact of the sound caused by the train passing by on the vibration response monitoring, a layer of PVC pipe was put around the sensor. All of the fiber optic accelerometers were connected by one optical cable with a spacing of about 6.5 m. The fiber optic phase interrogator was placed in a patrol house next to the high-speed railway to achieve the phase to vibration acceleration conversion and data storage. According to our previous research, the sensitivity of the fiber optic accelerometer reached up to 41 dB (re 0 dB = 1 rad/g) [21], and the noise level of each channel was below 12.6 ng/√Hz @ 5 Hz [24]. In this test, for more than one year, the sensor and the fiber optic phase interrogator were in good working condition, and the train-induced vibration response could be recorded in real time.

## 4. Results and Discussion

### 4.1. Selection of Feature Frequency Band

**Figure 4:**
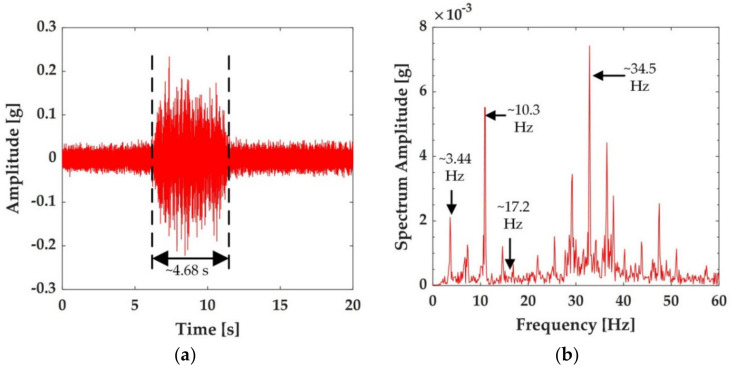
The vibration response of ballastless track recorded by the fiber optic accelerometer with train speed 310 km/h and track plate temperature 32 °C. (**a**) Time-history of ballastless track vibration accelerations. (**b**) Frequency spectra of ballastless track vibration accelerations.

Taking the CRH380AL (16 car formation) train as an example, in our experiment, when the train passed at 310 km/h on the ballastless track in the test section, the time-history of the vibration acceleration was completely recorded with fiber optic accelerometers, as shown in Figure 4a. The peak vibration induced by the train was about 0.24 g. Furthermore, it can also be observed from Figure 4a that the time taken for the train to pass the sensor location was about 4.68 s, which was consistent with the length of the CRH380AL train (403 m) and the speed of the train.

The time-history responses of the train-induced vibration acceleration shown in Figure 3a were converted to the amplitude spectra with a Fourier transformation to analyze the frequency–domain characteristics of the vibrations, as shown in Figure 4b. It can be observed from the figure that:

1. The significant frequency components in the spectra were spaced evenly, about 3.44 Hz, close to the integral multiple of the characteristic frequency (*f*_4_ = *v*/*L*_4_) induced by the center distance of two neighboring cars (25 m) of the CRH380AL train at the speed of 285 km/h;
2. The characteristic frequency *f*_2_ caused by the center distance of the adjacent bogies between the forward car and the backward *L*_2_ (7.5 m) was about 10.3 Hz, consistent with the dominant frequency of three-fold of *f*_4_;
3. The first dominant frequency was about 34.5 Hz, consistent with the characteristic frequency *f*_1_ caused by the wheelbase (2.5 m) and 10-fold of *f*_4_;
4. The dominant frequency of 5*f*_4_ = 17.2 Hz had zero amplitudes. This was due to the modulation amplitude effect caused by 3*f*_2_/2 and 7*f*_3_/2, which was consistent with the theoretical analysis of refs. [32,33] and the experimental result of ref. [25].

Based on the above analysis, the train-induced vibration acceleration of the ballastless track had an obvious response at frequency *nf*_4_. However, when *n* > 2, *f*_1_, *f*_2_, and *f*_3_ provided a modulation amplitude effect, which led to the complexity of the vibration response components at these frequencies. Therefore, the following analysis mainly focused on the train-induced vibration response characteristics of the ballastless track at the fundamental frequencies *f*_4_ and 2*f*_4_. For the running speed range from 120 km/h to 350 km/h, the fundamental frequencies *f*_4_ of the ballastless track vibration were in the range of 1.33–3.55 Hz. Because the sampling frequency of the vibration signal was 1 kHz, in order to obtain the highest possible frequency resolution of each package and to ensure that a single package covered the fundamental frequency *f*_4_ and 2*f*_4_ ranges, the decomposition level 6 was selected.

**Figure 5:**
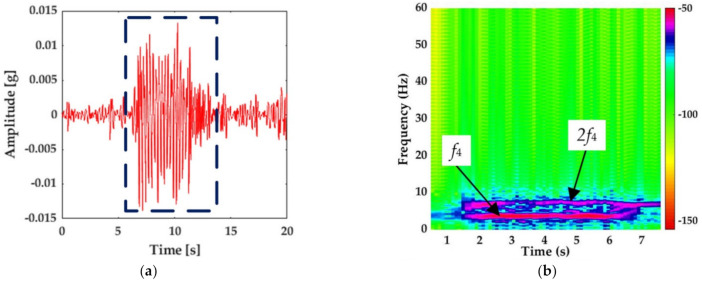
Reconstruction of vibration signal of characteristic frequency bands by the wavelet packet. (**a**) The time-history signal. (**b**) The time-frequency diagram.

Based on the above, the wavelet function, decomposition level, and the frequency parameters are shown in Table 1. The first frequency band of 0–7.82 Hz that covered the fundamental frequencies *f*_4_ and 2*f*_4_ was used as the characteristic frequency band of the WPT energy in the following analysis. The vibration response of the ballastless track slab recorded by the fiber-optic accelerometer shown in Figure 3 was taken as an example. The time-history signal of the characteristic frequency band after WPT is shown in Figure 5a, and its time-frequency diagram within 8 s is shown in Figure 5b. As can be seen from Figure 5, after the signal was reconstructed with WPT, the signals outside the characteristic band were removed. According to Formulas (1) and (2), the WPE of the characteristic frequency band was 3.57%.

### 4.2. WPT Energy of Vibration Response with Temperature-Induced Deformation

In the natural environment, ballastless track slab exchanges heat with the surrounding air medium through radiation, conduction, and convection, and its temperature field changes continuously with time and external temperature changes [4,5]. The temperature load caused by changes in natural conditions can be divided into the daily temperature load and the seasonal temperature load. In this section, first, the WPE change rule of the characteristic frequency band for the seasonal temperature load analysis is detailed and discussed. Then the WPE change rule of the characteristic frequency band for the daily temperature load analysis is detailed and discussed.

**Figure 6:**
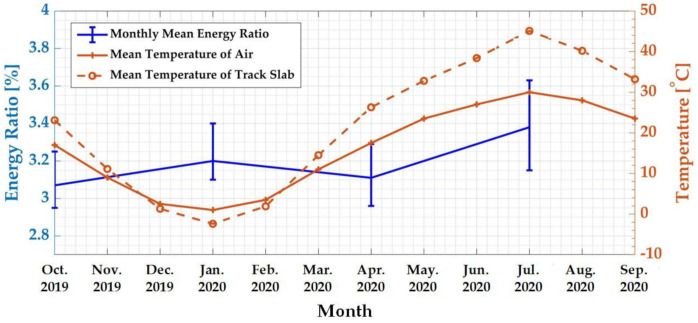
Monthly average wavelet packet transform (WPT) of characteristic frequency band and the monthly mean temperature.

The relationship between the monthly average WPE of the characteristic frequency band and the monthly mean temperature was analyzed. The results are shown in Figure 6. It can be observed that the air temperature changes of the experimental station and the surface temperature of the track slab in one year were periodic, with the lowest values in January and the highest values in July. Taking the most typical months of winter, spring, summer, and autumn as examples, which were January, April, July, and October, respectively, the monthly mean WPE of the characteristic frequency band was expressed as an error curve. Figure 6 shows that the WPE values in summer and winter were higher than those in spring and autumn, reaching 3.38% and 3.20%, respectively.

The reason for this phenomenon was that the track slab had the largest elongation and contraction in summer and winter. The temperatures in these two seasons were the highest and lowest in a year, respectively, which were the most unfavorable states for the concrete track slab structure [4]. The seasonal temperature change cycle was long, and the speed was slow, causing the ballastless track slab to produce longitudinal expansion and contraction deformation. Additionally, expansion and contraction stress occurred when the deformation was restrained. Under the action of temperature stress, the warpage of the track slab end and the horizontal and longitudinal displacements showed the characteristics of periodic expansion and contraction, which most often occurs in winter and summer. In winter, the temperature stress of the track slab was tensile stress, and the track slab exhibited a temperature shrinkage state. Due to the restraint of the CA mortar and its own temperature gradient stress, the track slab underwent temperature shrinkage, showing a concave shape with four corners that were upwardly warped. In summer, the temperature stress of the track slab was compressive stress, and the track slab showed a state of thermal expansion, showing a convex shape with four corners that were curved downwards [5,36]. Based on the above analysis, under the constraint of the adjacent track slab and the CA mortar layer, the deformations of the track slab were the largest in summer and winter. According to Figure 6, the WPE characteristic index values were also the largest in these two seasons, which was consistent with the track slab deformation law under a long-term seasonal temperature load.

**Figure 7:**
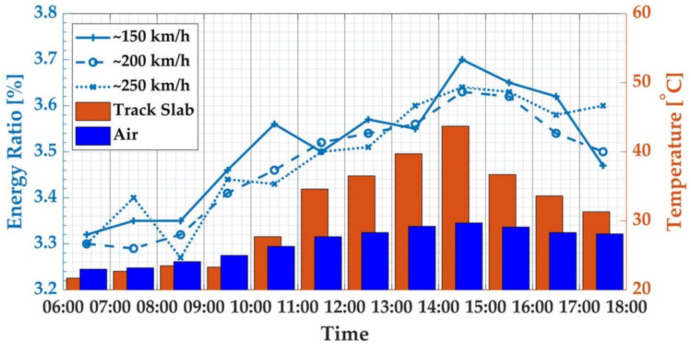
WPT of characteristic frequency band for 30 July 2020, summer.

We then analyzed the relationship between the WPE of the characteristic frequency band and the temperature in a day in summer. Figure 7 shows the WPE of the characteristic frequency band of the track slab vibration response caused by trains with three different speeds during the operation period of the high-speed railway on 30 July 2020. During testing, the surface temperature of the track slab changed synchronously with the air temperature, and both reached their peak values at 14:00–5:00. Due to the poor heat transfer performance of the concrete, the temperature difference between the upper surface and the lower surface of the track slab also reached the peak value during this period. At that time, the deformation of the track plate also reached the maximum value [10,11]. It can be seen from Figure 6 that the characteristic frequency-band energy ratio increased with the increase of the difference between the air temperature and the surface temperature of the track slab, and the characteristic frequency-band energy ratio reached the peak value in the period of 14:00–15:00.

**Figure 8:**
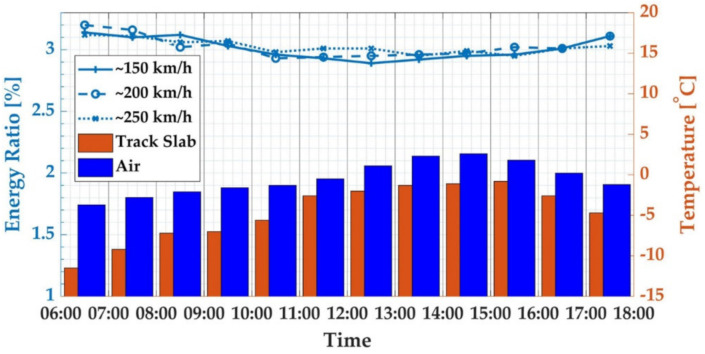
WPT of characteristic frequency bands for 16 January 2020, winter.

Figure 8 shows the WPE of the characteristic frequency band of the track slab vibration response caused by trains with three different speeds during the operation period of the high-speed railway on 16 January. The surface temperature of the track slab changed synchronously with the air temperature during testing, both reaching the peak values at 14:00–15:00. The peak temperature difference between the upper surface and the lower surface of the track slab appeared in the period of 06:00–07:00, and the difference decreased with the increase of the air temperature. During this period, the WPE of the characteristic frequency band was also the peak for the day.

According to Figure 7 and Figure 8, the WPE characteristic index change rule was different for a day in the summer and a day in the winter. The temperature change on the surface and the inside of the structure caused by the daily temperature load was a complex, random, non-linear function [37]. The surface of the track plate was in direct contact with the air, and the temperature rose rapidly under the action of solar radiation. The lower surface of the track plate was in close contact with the CA mortar layer, and the temperature was essentially unchanged under the action of solar radiation. Due to the low thermal conductivity of the concrete and the poor heat transfer performance, the rate of heat transfer from top to bottom was slow, so a temperature gradient was formed in the thickness direction, which caused the deformation of the track slab. Therefore, because the time period of the maximum longitudinal temperature difference of the track slab in winter and summer was different, the time when the maximum deformation occurred in one day was different. Similarly, in winter and summer, the time for the maximum WPE characteristic index in one day was different; it was consistent with the deformation characteristic of the track slab under the daily temperature load.

## 5. Conclusions

This paper proposed a long-term and real-time monitoring method for deformed track slabs based on WPE. Through the analysis of field test results in the Beijing–Shanghai High-speed railway, we found the maximum values of the WPE characteristic index appeared in winter and summer, and they were positively correlated with the temperature difference between the air environment and the track slab under daily temperature loads. This was consistent with the track slab deformation law under temperature loads. It was shown that the new method based on fiber-optic acceleration and WPE proposed in this paper could achieve long-term, real-time monitoring of track plate deformation. The result also implied that the proposed method was promising for the early warning of high-speed railway damage.

## Figures and Tables

**Table 1:** Wavelet function, decomposition level, and frequency parameters.

Parameter	Value
Wavelet function	Daubechies 6
Sampling frequency (Hz)	1000
Decomposition level *l*	6
Number of packets 2*^l^*	64
Frequency resolution of each packet (Hz)	7.82

## Data Availability

The data are not publicly available due to the Confidentiality and Non-disclosure Agreement with the funders.
